# Surgical debulking with fertility preservation in primary extra-neural ependymoma presenting with widespread peritoneal and intra-abdominal disease: Case report and literature review

**DOI:** 10.1016/j.gore.2026.102071

**Published:** 2026-04-01

**Authors:** Danielle Christmas, Harrison Odgers, Georgina Elton, Katrina Tang, Christine Kang, King Man Wan

**Affiliations:** a Department of Gynaecologic Oncology, Royal Hospital for Women, Barker Street, Randwick, NSW 2031, Australia; b School of Medicine, The University of Sydney, Camperdown, NSW 2050, Australia; c Prince of Wales Hospital, 320/346 Barker Street, Randwick, NSW 2031, Australia

**Keywords:** Ependymoma, Extra-neural ependymoma, Extra-axial ependymoma, Perivascular pseudorosettes, Fertility Preservation

## Abstract

**Background:**

Extra-neural ependymoma is an extremely rare neoplasm with reported cases arising from the ovary, pelvis, broad and uterosacral ligaments, peritoneum and mediastinum.

**Case Presentation:**

A 29-year-old woman presented with abdominal pain. Computed tomography demonstrated bilateral adnexal masses, omental and peritoneal nodules and a 140 mm left upper quadrant mass. FDG PET/CT showed avid, disseminated abdominal disease. CA125 was 265. Differential diagnoses included tubo-ovarian malignancy, peritoneal carcinomatosis, and mesenchymal tumours. Diagnostic laparoscopy with biopsy established Stage IV extra-neural ependymoma based on histological features, immunophenotype and the absence of central nervous system disease on magnetic resonance imaging. She underwent primary cytoreduction including en bloc hysterectomy with ultra-low anterior resection, right salpingoophorectomy, left salpingectomy, peritonectomy, splenectomy with en-bloc partial resection of the left hemi-diaphragm and loop ileostomy. Intraoperatively, the right ovary was utilised for ex-vivo in-vitro oocyte maturation, and the left ovary was conserved for oocyte retrieval and cryopreservation for future surrogacy. Adjuvant chemotherapy was recommended but declined. She remained well at eleven months post-operatively.

**Conclusion:**

Extra-neural ependymomas are exceedingly rare with five reported cases of peritoneal ependymoma. Extra-neural ependymomas demonstrate distinct histological and immunophenotypic profiles including increased architectural variability within the spindled cell perivascular pseudorosettes characteristic of ependymomas and diffuse ER and PR positivity. Given limited evidence, optimal management remains undefined however surgical cytoreduction continues to be the cornerstone of management across reported cases with adjuvant treatment individualised. Certain fertility preservation strategies are concurrently feasible within a multidisciplinary framework and should be considered given reported cases are predominantly seen in younger females.

## Highlights

• Extra-neural ependymomas predominantly affect young female patients, often presenting with large abdominal or pelvic masses.
• Extra-neural ependymomas show histological characteristics including perivascular pseudorosettes, increased architectural variability.
• Extra-neural ependymomas demonstrate immunohistochemical staining including estrogen and progesterone receptor positivity.
• Fertility preservation whilst still achieving optimal cytoreduction is feasible within a multi-disciplinary team.

## 1 Introduction

Ependymomas are rare low-grade neuroectodermal tumours which arise from glial ependymal cells lining the central nervous system (CNS). These tumours constitute 1.9% of central nervous system tumours with an incidence of 0.2–0.4 per 100,000 individuals (Vazzano et al., 2021 Nov). Extremely uncommonly, ependymomas may arise from extra-neural sites; most commonly from the sacrococcygeal region (Yust Katz et al., 2018), as well as from the ovaries (Kleinman et al., 1993), uterosacral and broad ligaments (Kumari et al., 2019), mediastinum and lungs (Zhou et al., 2015). Current treatment principles are largely extrapolated from intracranial and spinal ependymoma with reported cases undergoing cytoreductive surgery as primary treatment. We present a patient with an extra-neural ependymoma with widespread intra-abdominal and peritoneal disease who underwent primary cytoreductive surgery concurrently with fertility preservation treatment.

## 2 Case report

A 29-year-old woman with no medical, surgical or obstetric/gynaecological history presented with four days of abdominal pain, diarrhoea and anorexia. On examination her abdomen was moderately distended with suprapubic and right iliac fossa tenderness. Bimanual examination revealed right adnexal fullness and fixed uterus.

**Fig. 1:**
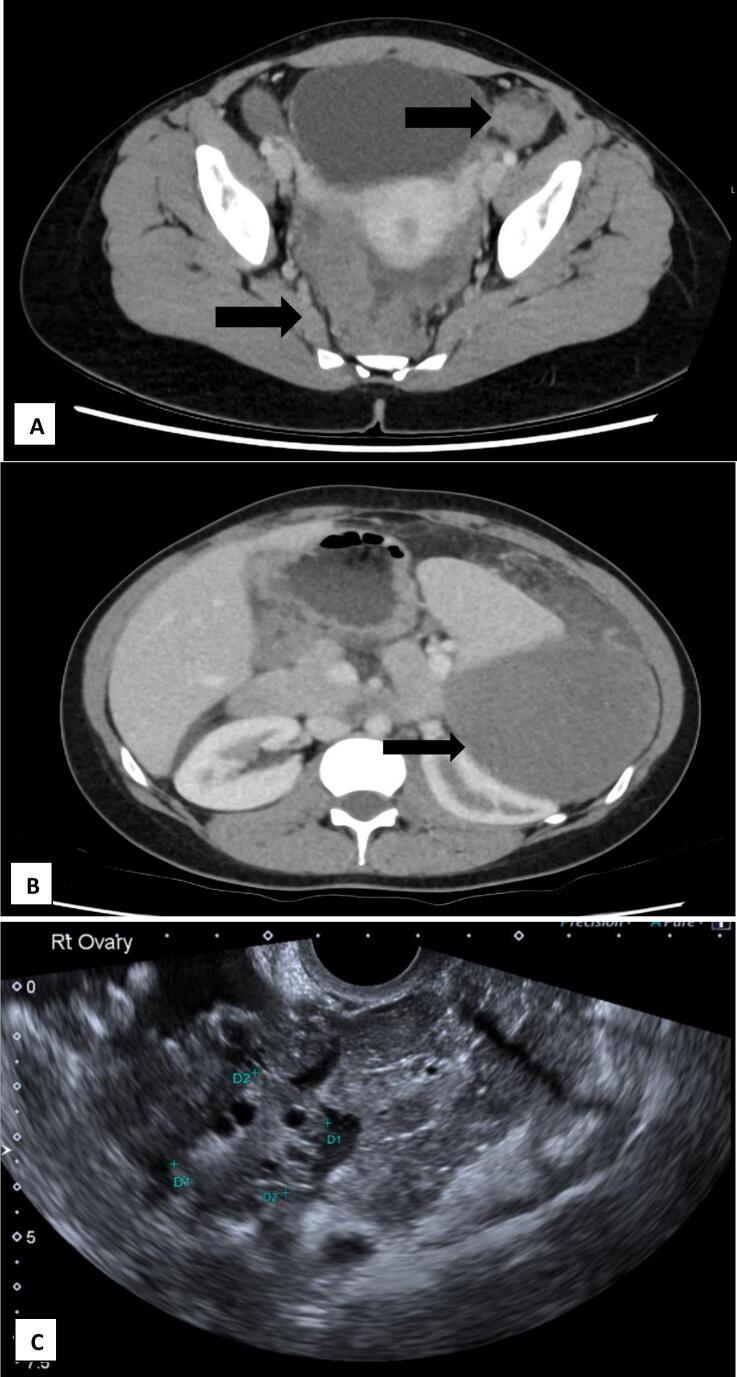
A- Axial view of pelvic CT scan demonstrating peritoneal nodules in the Pouch of Douglas, left lower quadrant nodular disease, ascites. B- Axial view of abdominal CT scan demonstrating large peritoneal mass in the left upper quadrant compressing the spleen and kidney. C- Sonographically normal right ovary surrounded by peritoneal nodules.

Full blood count, electrolytes/urea/creatinine and liver function tests were normal. CA125 was 265 U/mL and AFP, bHCG, CA19.9 and CEA were normal. Computed tomography of the chest, abdomen and pelvis (CT-CAP) demonstrated lobulated, heterogeneously enhancing bilateral adnexal masses (Fig. 1A). There were extensive omental and peritoneal nodules with a 140 mm mass in the left upper quadrant displacing the spleen (Fig. 1B). Ultrasound demonstrated vascular masses in both adnexa and peritoneum engulfing sonographically normal ovaries (Fig. 1C). A fluorodeoxyglucose positron emission tomography (FDG PET)/CT demonstrated avidity corresponding to peritoneal deposits along the right diaphragm, left upper quadrant, paracolic gutters, adnexae and pouch of Douglas.

A diagnostic laparoscopy, colonoscopy and gastroscopy showed ascites, an 80 mm solid lesion arising from the right fallopian tube, nodular tissue deposits over the right ovary, serosal surface of the uterus, and diffuse pelvic peritoneal nodularity involving the pouch of Douglas and rectum in a confluent plaque. There were multiple nodules over the peritoneum, omentum, sigmoid mesentery and diaphragms, and an enlarged spleen. Gastroscopy and colonoscopy were normal.

**Fig. 2:**
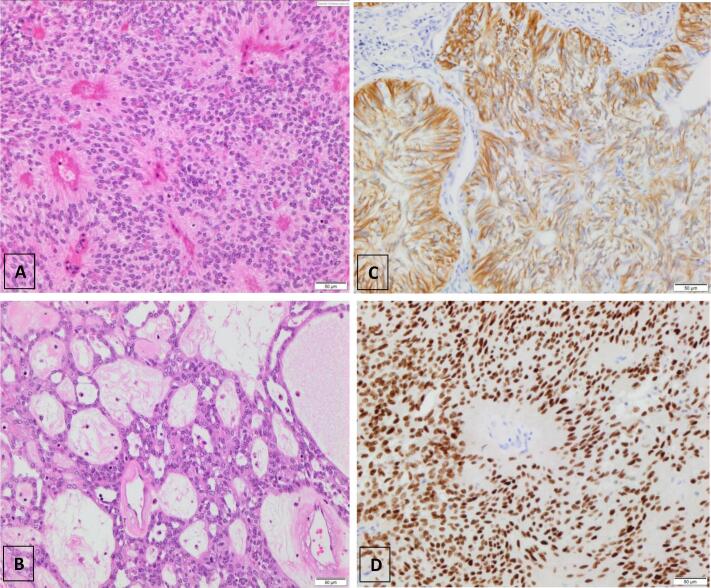
(all images taken at x200 magnification): A- H&E stain demonstrating pseudorosettes in tumour. B- H&E stain demonstrating areas of pseudoglandular architecture. C- GFAP immunohistochemistry was positive in tumour. D- PR showed strong diffuse nuclear staining (ER had a similar appearance).

Peritoneal and omental biopsies showed a tumour composed of spindled cells with striking perivascular pseudorosettes (Fig. 2A) composed of anuclear fibrillary material surrounding blood vessels. Areas of pseudopapillary and pseudoglandular architecture were seen (Fig. 2B). The tumour nuclei were oval with even chromatin. With immunohistochemistry, the tumour showed positive staining with glial fibrillary acidic protein (GFAP) (Fig. 2C), PAX8, WT1, EMA (luminal surfaces), cytokeratin and ER/PR (Fig. 2D). The morphology with positive GFAP and luminal EMA staining was consistent with ependymoma. Magnetic resonance imaging (MRI) confirmed no CNS lesions. The combination of the ER/PR positivity and absence of CNS lesions was consistent with a diagnosis of extra-neural ependymoma.

The patient was discussed at the Gynaecological Oncology Tumour board, and cytoreductive surgery was recommended. The patient was referred to the Oncofertility service for pre-operative assessment. Following multi-disciplinary discussion, the Gynaecological Oncology, Colorectal and Upper Gastrointestinal teams performed a midline laparotomy, en bloc hysterectomy with ultra-low anterior resection, right salpingoophorectomy and left salpingectomy, peritonectomy, splenectomy with en bloc partial resection of the left hemi-diaphragm and loop ileostomy formation (Fig. 5). The right ovary was sent for ex-vivo oocyte retrieval followed by in-vitro oocyte maturation. There was < 1 mm residual miliary disease on small bowel mesentery. Post-operative admission was 14 days complicated by a post-operative ileus requiring total parenteral nutrition through upper limb central venous catheter and an associated venous thromboembolism. She also experienced voiding dysfunction requiring intermittent self-catheterisation.

**Fig. 3:**
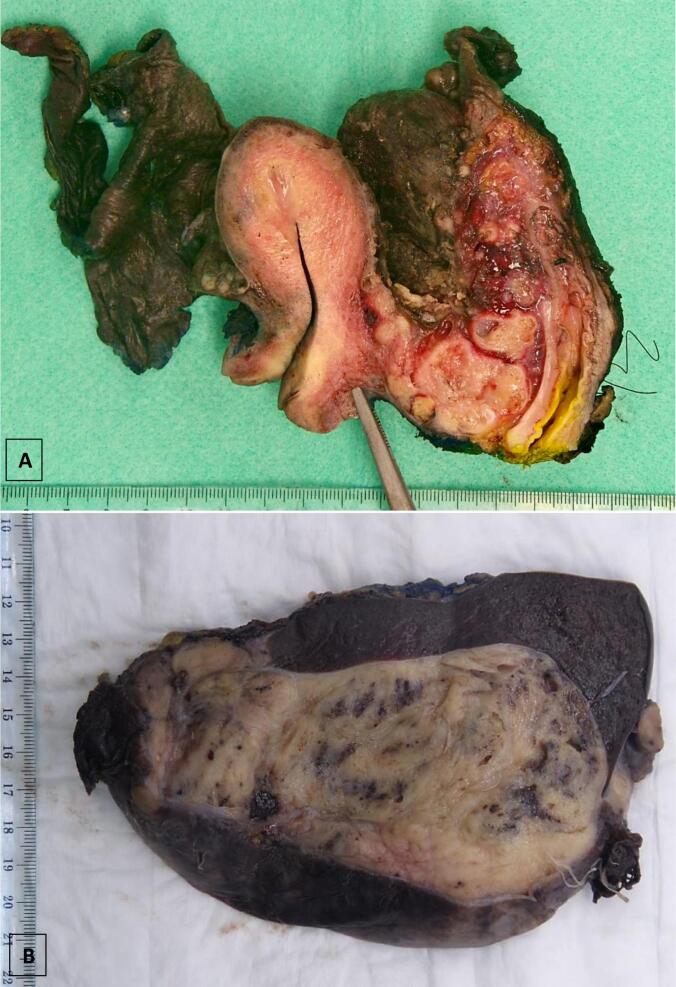
A- En Bloc resection of Pouch of Douglas tumour with uterus, rectosigmoid and right adnexa. B- Left upper quadrant tumour adjacent to spleen causing compression and displacement of spleen.

Histology confirmed extensive peritoneal involvement and a 100 mm pouch of Douglas mass invading into rectovaginal septum (Fig. 3A), 150 mm mass compressing splenic parenchyma (Fig. 3B), and an infarcted 82 mm right fallopian tube mass.

A diagnosis of Stage IV extra-neural ependymoma was assigned. Given the uncertainty regarding adjuvant treatment, expert opinion was sought internationally and from paediatric subspecialist teams. Ultimately, adjuvant etoposide and cisplatin were recommended however the patient declined. After recovery, the patient proceeded with two rounds of oocyte collection and subsequent ileostomy reversal. Serial CT-CAP demonstrated a sub-centimetre liver lesion, which was characterised on MRI as a 12 mm lesion concerning for metastatic disease. FDG-PET scan demonstrated no avidity within the liver. In the context of the indolent tumour nature described in the literature, a multidisciplinary consensus was reached for active surveillance with serial MRI and close clinical follow-up with consideration of further debulking surgery (including removal of left ovary due to ER positive status) at recurrence and completion of fertility preservation. Eleven months post diagnosis she remains well and asymptomatic of any disease.

## 3 Discussion

Primary extra-neural ependymomas are exceedingly rare tumours occurring outside the central nervous system, in contrast to classical ependymomas that originate from ependymal cells of the ventricles and spinal canal. Intra-abdominal extra-neural ependymomas have been most described as ovarian, with rare reports of uterine, mediastinal and peritoneal origin. In the current case, the primary tumour site was unable to be defined with certainty. Potential sites include the *peri*-splenic mass, pelvic soft tissue, or the right fallopian tube. The right ovarian surface was marginally involved and thus not suggestive of potential primary, while the left ovary appeared macroscopically normal and was not removed. Though it could not be confirmed by histopathology, the tumour distribution is consistent with previous cases of primary peritoneal extra-neural ependymomas whereby patients were found to have an abdominal or pelvic mass with widespread peritoneal involvement.

### 3.1 Distinguishing features of pelvic and peritoneal ependymomas

Our current case shares several clinical similarities to reported peritoneal and pelvic ependymomas. All cases of primary peritoneal and pelvic ependymoma (excluding sacrococcygeal origin) arose in females. This often occurred at a younger age than would be expected for primary tubo-ovarian or peritoneal malignancies. All patients initially presented with abdominal pain and bloating and were subsequently found to have a large abdominal, pelvic or peritoneal mass. Definitive diagnosis of primary extra-neural ependymoma requires histopathological examination of tumour tissue and exclusion of intracranial or spinal primary. Extra-neural ependymomas demonstrate characteristic histological and immunophenotypic features like those of neural ependymomas including the presence of pseudovascular rosettes and positive GFAP staining. Extra-neural ependymomas, however, do have some distinct morphological and immunohistochemical characteristics to their CNS counterparts. Morphologically, they demonstrate more architectural variability with our case demonstrating areas of both pseudopapillary and pseudoglandular architecture (Fig. 2B). Reported immunophenotypic differences include increased rates of positive expression of 34BE12, CK18, CK7, CAM 5.2 and notably extra-neural cases have demonstrated diffuse, strong staining for ER/PR in small case series (Idowu et al., 2008 May). These differences suggest that extra-neural ependymomas arise from distinct pathophysiological pathways.

### 3.2 Aetiology

Several theories are proposed to explain the origin of extra-neural ependymomas. Most cases arise close to the sacro-coccygeal region and filum terminale, leading authors to a germ cell origin theory whereby ependymomas originate from residual embryonic neuro-ectodermal tissue (ependymal rests) arising from remnants of the neuroenteric canal or of heterotopic ependymal cells with defects in the neural arch (Verdun and Owen, 2015). These rests have undergone incomplete regression or become displaced and subsequently undergo monodermal teratomatous proliferation (Vazzano et al., 2021 Nov). Primary ovarian extra-neural ependymomas, however, do not align with this as ependymal rests have not been found in ovaries. Pure ependymomas of the ovary are thought to represent a form of monodermal teratoma (Vazzano et al., 2021 Nov). Rare ovarian cases occurring as a component of an ovarian teratoma have also been reported (Gorski et al., 2017 May). Alternatively, neometaplasia of peritoneum and Mullerian duct tissue has also been proposed. There may also be a hormonal influence whereby misdirected primordial germ cells transform to ependymal cells under the influence of female hormones – supported by the female distribution and hormone receptor status seen in pelvic and peritoneal ependymomas (Yust Katz et al., 2018).

### 3.3 Imaging features

Imaging features of primary extra-neural ependymomas are rarely described and significantly overlap with those of primary ovarian malignancies. Some common characteristics include heterogeneously enhancing adnexal or pelvic masses and diffuse peritoneal metastases (Zhou et al., 2015). Invasion into adjacent structures is variable with cases arising from the broad ligament demonstrating local invasion into the adjacent bowel and inseparable from the uterus (Kumari et al., 2019).

There is very limited research on FDG-PET/CT characteristics with only one case whereby FDG-PET was used. Yamamoto et al reported a primary peritoneal ependymoma with low FDG avidity in tumour sites (SUVmax = 2.6). Contrarily, in our patient, the FDG-PET/CT demonstrated avid tumour with SUVmax of 7.2 in omental caking and peritoneal disease, 6.0 in the pouch of Douglas/pelvic mass and 5.1 in the splenic mass. Similar to CNS ependymomas, it is possible that FDG uptake in extra-neural ependymomas may depend on tumour grade and subtype (Zukotynski et al., 2014).

### 3.4 Treatment and prognosis

Due to the rarity of these tumours, there is no established gold standard treatment. Among published cases of peritoneal, pelvic and ovarian ependymomas, all other than one patient (Zhou et al., 2015) underwent primary surgery. In CNS ependymomas, surgery is the preferred treatment with total gross resection associated with improved survival. CNS ependymomas demonstrate radiosensitivity with adjuvant radiotherapy resulting in either no recurrence following total resection or stable disease in 52.9% of patients and either partial or complete response in a further 37.2% and significantly improved progression free survival (Zuccato et al., 2022 Aug 1). Radiotherapy is scarcely described in non-sacral extra-neural ependymomas potentially as disease is often widely disseminated throughout the abdomen and pelvis. There is a case of successful use of palliative radiotherapy for tumour recurrence in the lung in combination with oral etoposide resulting in reduction in tumour burden (Cheung et al., 2020).

Chemotherapy is also poorly understood with literature review of chemotherapy use in intracranial and spinal ependymomas yielding no evidence regarding chemotherapy in the adult adjuvant setting. In neural ependymomas, chemotherapy is generally reserved for salvage treatment of recurrence. Amongst patients with recurrence, a small retrospective study compared platinum and non-platinum based regimes and demonstrated a complete response rate of 15% and 0% respectively and a partial response rate of 15% and 13% respectively (Brandes et al., 2005). Additionally, bevacizumab has been used in recurrent disease with reported partial response rates in 6 of 8 patients with a median PFS of 6.4 months and OS of 9.4 months (Green et al., 2009). Amongst childhood ependymomas, active agents include cisplatin, carboplatin, cyclophosphamide and etoposide with multi-drug regimens demonstrating greater efficacy (Pan et al., 2025). From published case reports, in patients with extra-axial ependymoma where chemotherapy is given, most patients receive a multi-drug regime with platinum agent, etoposide with or without bleomycin.

Extra-neural ependymomas largely demonstrate positive estrogen and progesterone receptor staining, offering a potential therapeutic target. Zhou et al (Zhou et al., 2015) described a case in which primary treatment with leuprolide resulted in shrinkage of a pelvic extra-axial ependymoma facilitating surgery. There are also few cases of ovarian extra-neural ependymomas whereby aromatase inhibitors were used in the adjuvant or recurrent setting with stable disease over a period of follow up between 8––15 months (Gorski et al., 2017, Patel et al., 2020).

There are no reported cases whereby patients underwent fertility preservation treatment as part of or following their primary surgery. Our patient had ex-vivo retrieval of oocytes from the removed right ovary and two subsequent stimulated cycles of oocyte harvest from the remaining in-situ left ovary. As previously highlighted, extra-neural ependymomas are almost universally ER/PR positive and thus stimulation protocols may need to be carefully considered. This aspect of care is important to consider as many patients will be young females of child-bearing age and patients may have protracted survival even in the context of widespread disease.

Prognosis data for extra-neural ependymomas are limited, particularly for those outside the sacrococcygeal area. Several previous case reports demonstrate a slow, indolent growth of tumour with prolonged survival over years, even with metastasis. In previous case series, however, non-sacral ependymomas seemed to have a worse prognosis with a median survival of 10.95 years for non-sacral ependymomas and 27 years for sacrococcygeal ependymomas (Yust Katz et al., 2018).

## 4 Conclusion

Extra-neural ependymoma is an extremely rare neoplasm which has distinct characteristics compared to its CNS counterpart. It should be considered as an unlikely differential in young women presenting with disseminated abdominal and pelvic disease and diagnosed through its characteristic histopathology and immunohistochemical profile. Surgery remains the mainstay of treatment with the possibility of hormone inhibition requiring further investigation. Our case demonstrates the potential feasibility of fertility preservation options for primary debulk of this rare tumour with collaboration of the multi-disciplinary team and patient counselling. Our findings are limited by short period of follow up and thus further follow up is required to better understand the long-term outcomes following this treatment approach.

Written informed consent was obtained from the patient for publication of this case report and accompanying images.

## CRediT authorship contribution statement

**Danielle Christmas:** Writing – review & editing, Writing – original draft, Methodology, Investigation, Formal analysis, Conceptualization. **Harrison Odgers:** Writing – review & editing, Writing – original draft, Methodology, Investigation, Formal analysis, Conceptualization. **Georgina Elton:** Investigation, Formal analysis, Conceptualization. **Katrina Tang:** Writing – review & editing, Writing – original draft, Investigation, Formal analysis. **Christine Kang:** Writing – review & editing, Investigation, Data curation. **King Man Wan:** Writing – review & editing, Writing – original draft, Supervision, Methodology, Investigation, Data curation, Conceptualization.

## Declaration of competing interest

The authors declare that they have no known competing financial interests or personal relationships that could have appeared to influence the work reported in this paper.
